# Harnessing *Desmochloris edaphica* Strain CCAP 6006/5 for the Eco-Friendly Synthesis of Silver Nanoparticles: Insights into the Anticancer and Antibacterial Efficacy

**DOI:** 10.3390/molecules29163750

**Published:** 2024-08-07

**Authors:** Reham Samir Hamida, Mohamed Abdelaal Ali, Mariam Abdulaziz Alkhateeb, Haifa Essa Alfassam, Maha Abdullah Momenah, Mashael Mohammed Bin-Meferij

**Affiliations:** 1 Institute for Protein Research, Osaka University, Osaka 565-0871, Japan; 2 Plant Production Department, Arid Lands Cultivation Research Institute, City of Scientific Research and Technological Applications (SRTA-CITY) New Borg El-Arab, Alexandria 21934, Egypt; 3 Department of Biology, College of Science, Princess Nourah bint Abdulrahman University, Riyadh 11671, Saudi Arabia

**Keywords:** biological synthesis, nanoparticles, optimization, cancer, bacteria

## Abstract

Microalgae-mediated nanoparticle (NP) biosynthesis is a promising green synthesis method that overcomes the challenges of conventional synthesis methods. The novel *Desmochloris edaphica* strain CCAP 6006/5 was isolated, purified, and characterized morphologically and genetically. GC-MS analysis of the algal biomass (D_Bio_) phytochemicals showed the abundance for elaidic acid (18.36%) and monoolein (17.37%). UV-VIS spectroscopy helped analyze the effects of the AgNO_3_ concentration, algal/silver nitrate ratio, temperature, reaction time, illumination, and pH on AgNP synthesis. D_Bio_ extract or cell-free medium (D_Sup_) of *D. edaphica* successfully biosynthesized small silver NPs (AgNPs), namely, D_Bio_@AgNPs and D_Sup_@AgNPs, under optimum reaction conditions. TEM and SEM showed a quasi-spherical shape, with average diameters of 15.0 ± 1.0 nm and 12.0 ± 0.8 nm, respectively. EDx and mapping analyses revealed that silver was the main element, the NP hydrodynamic diameters were 77.9 and 62.7 nm, and the potential charges were −24.4 and −25.8 mV, respectively. FTIR spectroscopy revealed that the D_Bio_@AgNPs, and D_Sup_@AgNPs were coated with algal functional groups, probably derived from algal proteins, fatty acids, or polysaccharides, representing reductant and stabilizer molecules from the synthesis process. They showed significant anticancer activity against breast cancer cells (MCF-7), low toxicity against normal kidney cells (Vero), and potent inhibitory activity against *Staphylococcus aureus*, *Bacillus subtilis*, and *Shigella flexneri*. *D. edaphica* is a novel biomachine for synthesizing small, stable and potent therapeutic AgNPs.

## 1. Introduction

Owing to their superior biological and physicochemical properties, silver nanoparticles (AgNPs) have been used in a wide range of industrial, medicinal, and agricultural applications [1,2,3]. AgNPs are currently used in healthcare products and cosmetics. They are used in food packaging to increase the shelf life of foods [3,4]. Owing to their high reactivity, they have been used as potent anticancer agents against colon, liver, breast, and other cancers [5,6]. Additionally, they exhibit excellent biocidal activity against bacteria, fungi, and viruses [7,8,9]. Despite these significant applications of AgNPs, fabricating safe and biocompatible nanoparticles (NPs) for absolute usage remains a major issue. AgNP accumulation in the gastrointestinal tract may aggravate injuries such as abnormality of the epithelial cell microvilli and intestinal glands and an inflammatory response in intestinal native colonies. After gastrointestinal absorption of AgNPs, Ag ions accumulate in organs such as the liver, spleen, and kidney, which may cause undesirable health effects [3,10]. Recently, nanobiotechnology has enabled novel green synthesis approaches to produce AgNPs that are small, stable, and highly biocompatible compared with those synthesized using conventional methods [11,12]. In the green synthesis method, living cells and their purified molecules are the primary sources for reducing and stabilizing NPs [12]. These natural resources exist in the environment and can be readily obtained and cultured. For instance, plants, algae, fungi, bacteria, lichens, and actinomycetes and their purified molecules, such as proteins and polysaccharides, are potent biomaterials for NP synthesis [13,14]. This requires no toxic chemicals for the synthesis reaction; consequently, few to no hazardous compounds are transferred to the environment and other living organisms. Another advantage of green synthesis is the feasibility of coating the resulting NPs with many functional groups that occur in natural biomolecules, such as proteins, lipids, pigments, antioxidants, and fatty acids. These coronae impart the NPs with several advantages, such better stability in various salt-containing solutions and enhanced biocompatibility and selectivity than chemically or physically synthesized NPs [15,16]. Recently, microalgae have been considered one of the superior biofactories for NPs owing to their potential to detoxify and accumulate heavy metals from the environment, rapid growth, high yield, and ease of culture under normal environmental conditions [17]. The potential of microalgae to reduce precursors into their nanoforms continues to be investigated. Microalgae are enriched with many vital biomolecules, such as unsaturated fatty acids, antioxidants, vitamins, peptides, and phenolics [18]. Thus, NPs may be coated with these potent biomolecules, which may act as targeting ligands for cell receptors, therapeutic agents, or oxidative stress inhibitors [19]. Additionally, NPs have been produced in several shapes (cubic, spherical, triangular, and hexagonal) and sizes using microalgae. These biogenic NPs show high bioactivity against malignant and microbial cells and low toxicity against normal cells [12,20]. A significant factor in obtaining small and stable NPs using green synthesis methods is the optimization of the reaction parameters, such as the reactant concentration, pH, temperature, reaction time, and light [21]. In this study, we demonstrate for the first time the potential of the novel *Desmochloris edaphica* strain CCAP 6006/5 to synthesize AgNPs using two methods: one with a cell-free medium and another with cell biomass extract. We optimize the reaction conditions for synthesizing small AgNPs using the tested microalgae. To the best of our knowledge, this is the first study to demonstrate the presence of volatile biomolecules in the novel microalgae. The AgNPs are physicochemically characterized and applied against malignant and normal cells and Gram-positive and Gram-negative bacteria.

## 2. Results and Discussion

### 2.1. Algal Identification

**Figure 1:**
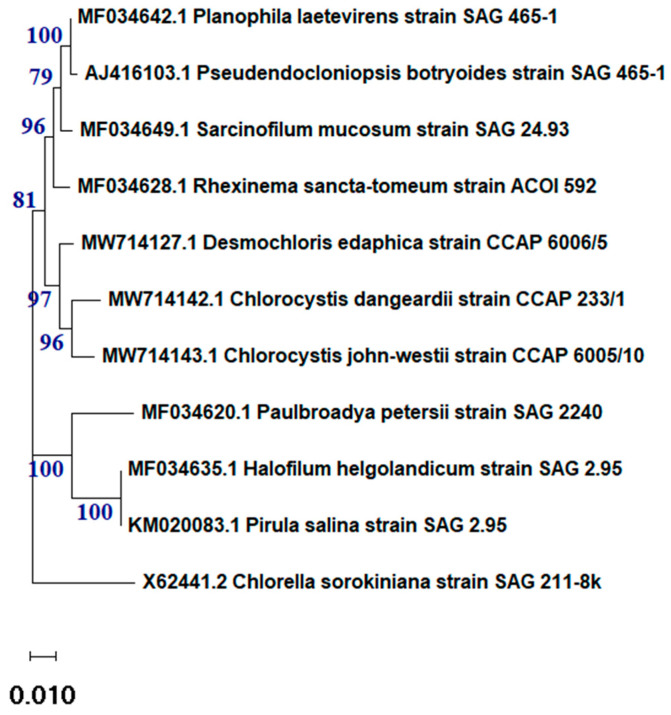
The phylogenetic tree of *Desmochloris edaphica* strain CCAP 6006/5 was inferred from the 18S rRNA and constructed using the cluster method with MEGA4 software version 10.2.6.

**Figure 2:**
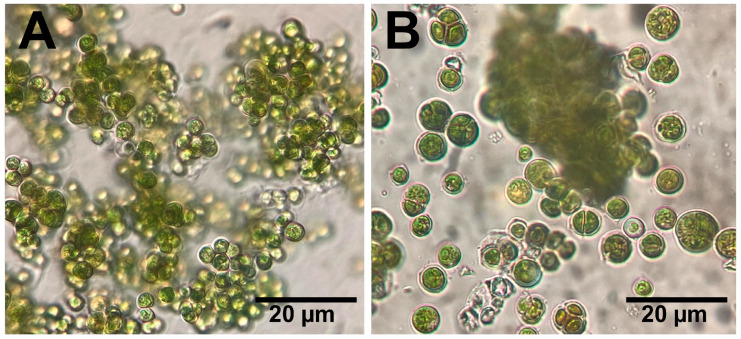
Light micrographs (**A**,**B**) reveal the morphological appearance of *D. edaphica* strain CCAP 6006/5. Scale bar 20 µm.

The data analysis of the 18s rRNA sequence of the algal isolate showed that the sample was 97.8% identical to *Desmochloris edaphica* strain CCAP 6006/5 (Figure 1). The sequence was deposited in the NCBI website collection (accession number: OR826101). The morphological appearance of *D. edaphica* strain CCAP 6006/5 was examined using light microscopy (Figure 2). The micrographs illustrate that the sample presented a spherical shape, had an olive-green color, and formed clumps. They were surrounded by thin cell walls and contained chloroplasts with one large visible pyrenoid. Vegetative cells were either solitary or in packages. These data are consistent with those of Darienko et al., who identified *Desmochloris edaphica* CCAP 6006/5 genetically and morphologically using the SSU-ITS sequences and light microscopy, respectively. They found that the new species was spherical, with a diameter of 6.2–9.9 µm [22]. These single cells were surrounded by thin walls and had cup-shaped chloroplasts with 2–3 incisions covering almost the entire cell. A large pyrenoid surrounded by many starch grains was detected in the chloroplasts, whereas a large vacuole was detected in the young cells. Mature vegetative cells were spherical and existed either solitarily or in packages of 2–8 cells.

**Figure 3:**
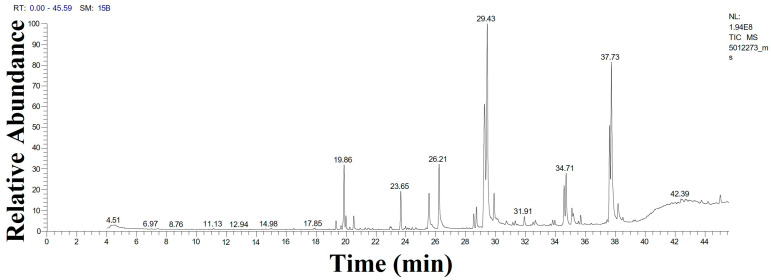
GC-MS chromatogram illustrating the compositions of the volatile phytochemicals detected in the *D. edaphica* strain CCAP 6006/5 biomass extract.

Gas chromatography–mass spectrometry (GC-MS) was used for the first time to detect volatile molecules in the aqueous extract of *D. edaphica* biomass (Figure 3 and Table 1). Elaidic acid (18.36%) was the most abundant fatty acid in the sample, followed by monoolein (17.37%), linoleic acid (12.83%), 2-monolinolein (9.24%), and palmitic acid (5.95%). Moreover, some aromatic monoterpenoids, such as acetic acid and [1-(4-isopropylphenyl)-2-methyl] propyl ester (6.6%), were also detected. This result highlights the importance of *D. edaphica* as an enriched source of unsaturated fatty acids for the food and pharmaceutical industries. Monoolein is considered an important fatty acid for drug delivery [23]. Additionally, these dominant fatty acids may be the primary stabilizing agents for AgNPs. 

### 2.2. AgNPs Synthesis Using Desmochloris Edaphica Strain CCAP 6006/5

#### Characterization

The precursor concentration significantly influenced the synthesis of D_Bio_@AgNPs and D_Sup_@AgNPs. The data demonstrate that an increase in the AgNO_3_ concentration from 1 to 2 mM caused a blueshift in the NP wavelength from 451 to 422 nm for D_Bio_@AgNPs and from 440 to 414 nm for D_Sup_@AgNPs. Additionally, changing the AgNO_3_ concentration from 2 to 4 or 8 mM caused a redshift in the NP wavelength or did not form NPs. These results showed that the optimal AgNO_3_ concentration for synthesizing small D_Bio_@AgNPs and D_Sup_@AgNPs was 2 mM. Deviation from the optimal concentration of AgNO_3_ either promotes the development of NP clusters or prevents the formation of NPs. As the concentration of AgNO_3_ was increased while maintaining a constant volume of algal extract, the quantity of reductants was inadequate for facilitating the reduction of AgNO_3_ to AgNPs. According to Sobczak-Kupiec et al., an increase in the concentration of AgNO_3_ leads to the formation of larger-sized NPs and increases the aggregation of NPs [24].

Similarly, changing the algal extract/AgNO_3_ (V_mL_/V_mL_) ratio from 1:2A to 1:1, 1:4A, 1:9A, 1:2B, 1:4B, and 1:9B influenced the particle size. The optimum ratio for synthesizing small D_Bio_@AgNPs was 1:9A at 414 nm, whereas the optimum ratio for synthesizing small D_Sup_@AgNPs was 1:2A at 414 nm. Changing this ratio by increasing the volume of the algal extract or precursor resulted in the formation of larger or aggregated NPs. We conclude that the size of AgNPs is highly affected by the ratio between the reductants (algal biomolecules) and the precursor, as well as the precursor concentrations. Therefore, it can be inferred that the change in the AgNO_3_ concentration depends on the reducing agent concentration in order to achieve a balanced synthesis reaction [19]. Haji et al. showed that an increase in the AgNO_3_ concentration increased the average particle size [25].

The influence of temperature on the synthesis of D_Bio_@AgNPs and D_Sup_@AgNPs was investigated. The results showed that the optimum temperature for synthesizing D_Bio_@AgNPs and D_Sup_@AgNPs was 25 and 60 °C at 414 and 404 nm, respectively. Notably, an increase in the optimum reaction temperature resulted in a redshift of the NP wavelength, indicating the formation of unstable or large AgNPs. Moreover, the synthesis of D_Bio_@AgNPs at 100 °C resulted in a pale-yellow solution, indicating an incomplete synthesis reaction at 404 nm [26]. The formation of D_Bio_@AgNPs and D_Sup_@AgNPs at a certain temperature might be attributed to the metabolic activity of the algal extract. The formation of larger-sized D_Bio_@AgNPs at increased temperatures could be attributed to the response of algal biomolecules (reductants or stabilizers) to high temperatures, which could cause their inactivation, or denaturation. This could cause an unbalanced redox reaction, leading to the formation of unstable AgNPs or aggregates. Notably, D_Sup_@AgNPs synthesis suggested that temperature is a significant factor in activating the D_Sup_@AgNPs synthesis reaction. However, this could be attributed to the fact that the biomolecules responsible for the synthesis of D_Bio_@AgNPs and D_Sup_@AgNPs may not have the same chemical compositions and chemical behavior. A surge in the reaction temperature accelerated the formation of Ag clusters and decreased the AgNO_3_ concentration. Hence, uniform AgNPs were formed at a rapid reduction rate [27]. To sum up, at the optimum reaction temperature, the algal biomolecules become active, while increasing the temperature beyond that point could result in degradation, denaturation, or deactivation of biomolecules that are responsible for reducing and stabilizing NPs and the production of agglomerated AgNPs or an incomplete synthesis reaction [28]. Lengke et al. studied that the shape of AgNPs synthesized by *Plectonema boryanum* under-influenced the reaction temperature change. A spherical shape of AgNPs was dominant at 25–100 °C, whereas only octahedral shapes appeared at 100 °C [29]. Prasad et al. reported that the reaction temperature influenced the NPs’ size. The scholars found that the size of AgNPs synthesized by *Cystophora moniliformis* at a lower temperature than 65 °C was 75 nm, whereas NPs self-assembled and formed clusters at a temperature higher than 95 °C with a size of 2 µm [30]. Liu et al. found that the formation of NPs at higher temperatures may be because of an increase in the nucleation kinetics constant rather than a decrease in the growth kinetics constant [31].

To analyze the influence of the reaction time, D_Sup_@AgNPs were synthesized at the optimal temperature (60 °C) for 15, 30, and 60 min. The data showed that increasing the reaction time from 15 to 30 or 60 min resulted in no change in the NP wavelength (407 nm). In general, an increase in the reaction time leads to an increment in the number of synthesized NPs, but only for a certain amount of time. Following this point, there might be an agglomeration of AgNPs due to their instability [32]. These data indicate that if the D_Sup_@AgNPs were stable, there would be no effect of an increasing time on them. Exposure to illumination strongly influenced the synthesis of D_Bio_@AgNPs and D_Sup_@AgNPs. The synthesis of D_Bio_@AgNPs and D_Sup_@AgNPs under dark conditions did not form D_Bio_@AgNPs and caused a redshift in the wavelength of D_Sup_@AgNPs from 404 nm (under light exposure) to 430 nm (under no light exposure). These data suggest that biofabrication of AgNPs using algal extracts is a photoreduction process in which light energy plays an important role in activating the synthesis reaction for both D_Bio_@AgNP and D_Sup_@AgNP fabrication [19,33]. Patel et al. synthesized AgNPs using *Scenedesmus* sp. under dark and light conditions. The scholar found that AgNPs synthesized only under light conditions, suggesting the important role of light in the reduction reaction of AgNPs [34].

**Figure 4:**
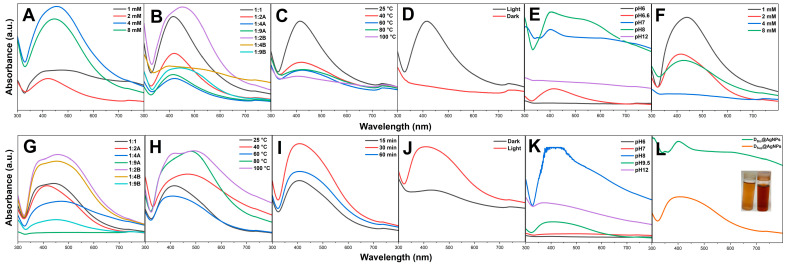
Wavelengths of D_Bio_@AgNPs (**A**–**E**) and D_Sup_@AgNPs (**F**–**K**) synthesized using *D. edaphica* biomass extract and cell-free medium under various reaction conditions, including the reactant concentration (**A**,**F**), algal/AgNO_3_ ratio (V_mL_/V_mL_ (**B**,**G**)), temperature (**C**,**H**), reaction time (**I**), illumination (**D**,**J**), and pH (**E**,**K**). (**L**) represent the wavelengths of D_Bio_@AgNPs (golden NPs suspension) and D_Sup_@AgNPs (reddish-brown NPs suspension) after adjusting all the optimum conditions.

Changing the pH of the D_Bio_@AgNP synthesis reaction from the initial pH (6.6) to 6 and 12 did not form AgNPs, whereas changing the initial pH to 7 and 8 resulted in a blueshift in the wavelength of D_Bio_@AgNPs from 414 nm to 401 and 402 nm, respectively. The optimum pH for the synthesis of D_Sup_@AgNPs was the initial pH 9.5, and changing this pH to acidic or basic conditions enhanced the aggregation of D_Sup_@AgNPs. Gontijo et al. found that AgNPs were less aggregated at pH < 3 and > 7 because all the groups were protonated at pH < 3 and working against electrostatic interactions, whereas at pH > 7, all the groups were deprotonated, which favored repulsion between the NPs and discouraged their agglomeration [35]. To sum up, the optimum synthesis parameters for D_Bio_@AgNPs and D_Sup_@AgNPs are as follows: D_Bio_@AgNPs: mixing 1 mL algal extract and 9 mL of 2 mM AgNO_3_ at 25 °C under illumination and pH 7 for 24 h; D_Sup_@AgNPs: mixing 1 mL algal extract with 2 mL of 2 mM AgNO_3_ at 60 °C for 1 h under illumination and pH 9.5 for 24 h (Figure 4).

**Figure 5:**
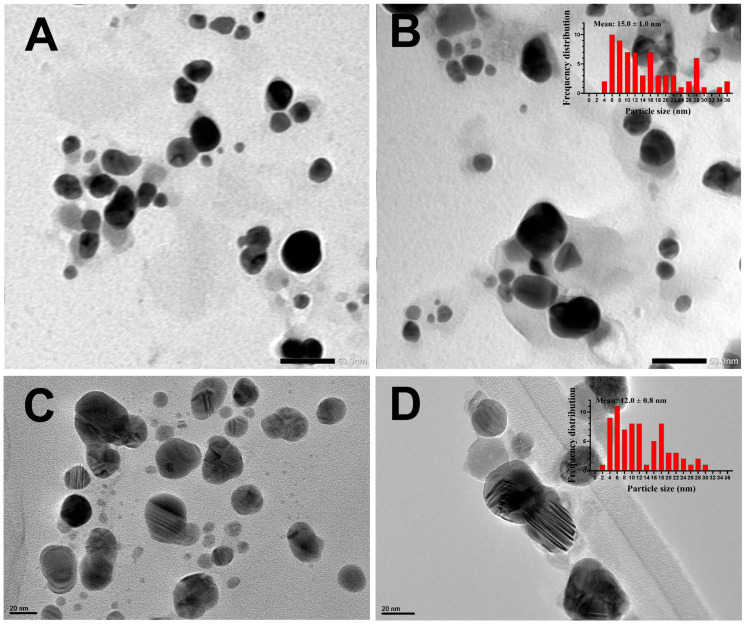
Transmission electron microscopy (TEM) micrographs with frequency distribution histograms demonstrating the shapes and sizes of D_Bio_@AgNPs (**A**,**B**) and D_Sup_@AgNPs (**C**,**D**) synthesized using *D. edaphica* biomass extract and cell-free medium. Scale bar of (**A**,**B**) is 50 nm and of (**C**,**D**) is 20 nm.

To study the shape and size of D_Bio_@AgNPs and D_Sup_@AgNPs, the samples were examined using TEM (Figure 5). The data showed that D_Bio_@AgNPs and D_Sup_@AgNPs were uniformly distributed, with no agglomeration. D_Bio_@AgNPs and D_Sup_@AgNPs presented a quasi-spherical shape, but D_Bio_@AgNPs had a few triangular NPs. The average diameters (nm) of D_Bio_@AgNPs and D_Sup_@AgNPs were 15.0 ± 1.0 nm and 12.0 ± 0.8 nm, respectively. Additionally, the TEM micrographs revealed the presence of algal organic matrix-coated NPs. These data have clarified for the first time the potential of *D. edaphica* CCAP 6006/5 to biofabricate silver nitrate into small AgNPs. However, our data were similar to the data reported by Pernas-Pleite et al., who synthesized AgNPs using a broth medium of unclassified microalgae isolated from the Tinto river estuarian waters [36]. They investigated the influence of the broth pH, media composition, and growth phase on AgNP synthesis. They found that these NPs had a quasi-spherical shape and a size range of 7–11 or 12–21 nm, respectively, depending on the absence and presence of chloride ions. Similarly, the AgNPs synthesized using *Chlorococcum humicola* presented a spherical shape and a nanosize range of 2–16 nm [37].

**Figure 6:**
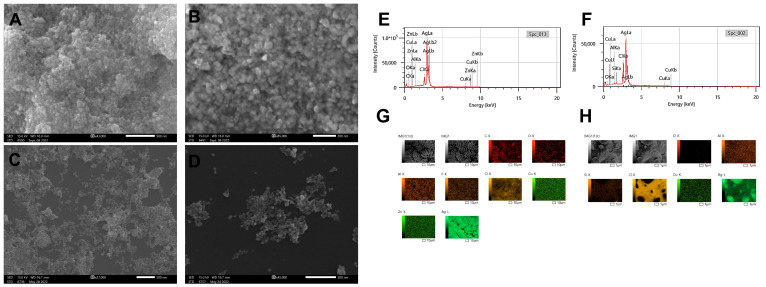
Scanning electron microscopy (SEM) micrographs of D_Bio_@AgNPs (**A**,**B**) and D_Sup_@AgNPs (**C**,**D**) synthesized using *D. edaphica* biomass extract and cell-free medium. EDx (**E**,**F**) and mapping analysis (**G**,**H**) illustrating the elemental composition and distribution of D_Bio_@AgNPs and D_Sup_@AgNPs, respectively. Scale bar of (**A**,**C**,**D**) is 500 nm and of (**B**) is 200 nm. Scale bar of (**G**,**H**) are 10 µm and 1 µm, respectively.

Similar to the TEM micrographs, the SEM micrographs confirmed that the D_Bio_@AgNPs and D_Sup_@AgNPs had quasi-spherical shapes (Figure 6). To determine the elemental composition and distribution of the D_Bio_@AgNPs and D_Sup_@AgNPs, the samples were scanned using an EDx detector conjugated to the SEM. The main element detected in D_Bio_@AgNPs and D_Sup_@AgNPs was silver with a mass % of 83.81 ± 2.29% and 85.15 ± 0.11%, respectively (Table 2 and Figure 6). Trace amounts of other elements, including carbon, oxygen, chloride, zinc, copper, phosphorus, aluminum, and silica, were found. The presence of carbon, oxygen, chloride, zinc, copper, and phosphorus indicates the successful synthesis of AgNPs using *D. edaphica*. These elements are the major minerals present in algal nutrients and their bioprocess [38]. Aluminum and silica were found in small quantities of 0.2% and 0.6%, respectively, and could have been included because of the sample preparation method. These results are in agreement with those of Chokshi et al., who performed SEM-EDx analysis of AgNPs synthesized using *Acutodesmus dimorphus*. They found that 76% of the sample weight was silver, with the strongest peak at 3 keV. These data were confirmed through mapping analysis [39].

**Figure 7:**
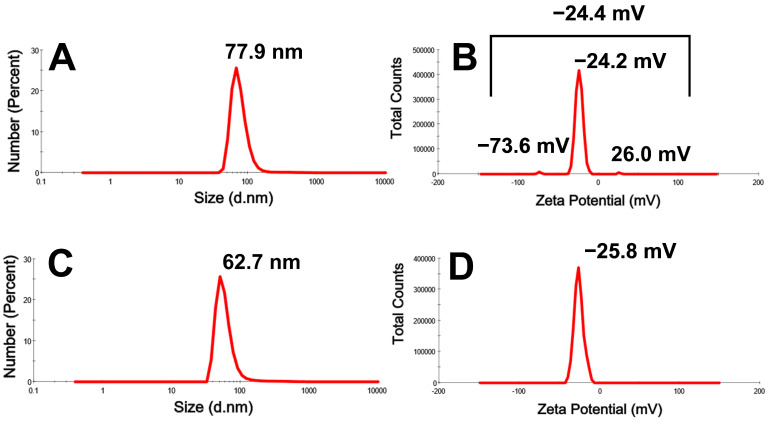
Hydrodynamic diameter (nm) and zeta potential (mV) of D_Bio_@AgNPs (**A**,**B**) and D_Sup_@AgNPs (**C**,**D**) synthesized using *D. edaphica* biomass extract and cell free medium, respectively.

The hydrodynamic diameters (HDs) and potential charges of D_Bio_@AgNPs and D_Sup_@AgNPs were examined using a Zetasizer (Figure 7). The HDs of D_Bio_@AgNPs and D_Sup_@AgNPs were 77.9 and 62.7 nm, respectively, whereas their charges were −24.4 and −25.8 mV, respectively. The electronegativities of D_Bio_@AgNPs and D_Sup_@AgNPs could be related to the algal functional groups on their surfaces. Cekuolyte et al. synthesized AgNPs using various strains of *Geobacillus* spp. (encoded by residues 18, 25, 95, and 612). The authors reported that the HDs of all the AgNPs were <100 nm, with a zeta potential of −26.6 ± 0.5, −31.3 ± 0.8, −25.7 ± 0.8, and −27.4 ± 0.6 mV, respectively [40].

**Figure 8:**
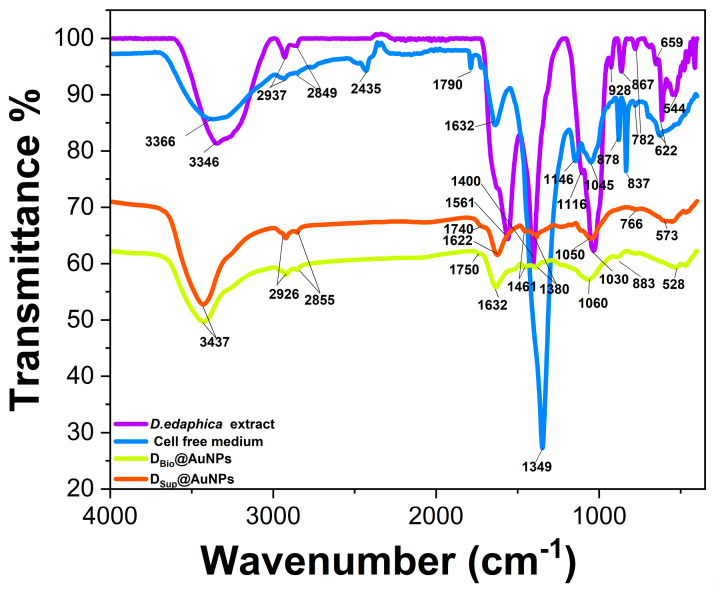
Fourier transform infrared spectroscopy (FTIR) analysis of the algal biomass extract, cell-free medium, D_Bio_@AgNPs, and D_Sup_@AgNPs, illustrating the chemical composition of the NP surfaces.

FTIR analysis was performed to study the surface chemistry of the novel D_Bio_@AgNPs and D_Sup_@AgNPs and the interaction of the algal biomolecules with the AgNP surfaces. The IR spectra of the *Desmochloris edaphica* biomass extract, supernatant, D_Bio_@AgNPs, and D_Sup_@AgNPs are presented in Figure 8 and Table 3. We found that O–H stretching existed in the *D. edaphica* biomass extract, supernatant, D_Bio_@AgNPs, and D_Sup_@AgNPs at 3346, 3437, 3366, and 3437 cm^−1^, respectively. Both D_Bio_@AgNPs, and D_Sup_@AgNPs have the same wavenumber, suggesting that the O–H group was very important for the AgNP synthesis process. Similarly, C–H stretching was detected in the *D. edaphica* biomass extract and supernatant at 2937 and 2849 cm^−1^, and shifted to be at 2926 and 2855 cm^−1^ in the D_Bio_@AgNPs and D_Sup_@AgNPs. These data suggested that an aliphatic chain coats the NPs’ surface and could play an important role in stabilizing the NPs. C=O stretching was detected in the supernatant, D_Bio_@AgNPs and D_Sup_@AgNPs at 1790, 1750 and 1740 cm^−1^, respectively, suggesting that aldehyde or esters could enhance the formation of NPs. N–H/C=C stretching was detected in the *D. edaphica* biomass extract, supernatant, D_Bio_@AgNPs, and D_Sup_@AgNPs at 1561, 2435/1632, 1632 and 1622 cm^−1^, respectively. This shift in the IRs’ wavelength and change in their intensities suggest successfully coating the NP surfaces with protein- or nitrogen-containing compounds. C–H bending was detected in the *D. edaphica* biomass extract, supernatant, D_Bio_@AgNPs, and D_Sup_@AgNPs at 1400, 1349, and (1461 and 1380) cm^−1^, respectively, suggesting the existence of organic molecules such as fatty acids. C–O stretching was detected in the *D. edaphica* biomass extract, supernatant, D_Bio_@AgNPs, and D_Sup_@AgNPs at 1116 and 1030, 1146 and 1045, 1060 and 1050 cm^−1^, respectively, suggesting that polysaccharides, alcohol or phenol or ester act as reactants during the synthesis process. C–H binding was detected in a range of 928–766 cm^−1^ in all the samples, indicating the existence of alkanes on the surfaces of NPs. C–Br stretching was detected in all the samples in the 528 to 659 cm^−1^ range. Interestingly, the number of IR peaks in the fingerprint area in the case of the *D. edaphica* biomass extract and supernatant was more than that in the case of D_Bio_@AgNPs, and D_Sup_@AgNPs, referring to the selectivity properties of the biological synthesis method in which specific hydrocarbons are responsible for the stabilization. To summarize, the functional groups present in the *D. edaphica* biomass extract and supernatant included O–H, C–H, C=C/N-H, C–O, C=O, and C–Br, suggesting the presence of organic molecules such as proteins, fatty acids, and polysaccharides. Although there were common functional groups between the *D. edaphica* biomass extract and supernatant, including O–H, C–H, C=C/N–H, C–O, and C–Br still, there were differences in the IR wavenumber, intensities, and the existence of new IRs such as C=O in the supernatant.

D_Bio_@AgNPs and D_Sup_@AgNPs had functional groups similar to those in the *D. edaphica* biomass extract and supernatant, such as O–H, C–H, C=C/N–H, C–O, C=O, and C–Br. The IRs of these functional groups were slightly shifted and had different intensities compared with the IRs of the *D. edaphica* biomass extract and supernatant. These data suggest that AgNPs were synthesized because of the bioreducing properties of the *D. edaphica* extracts. The existence of O–H, N–H, C–O, C–H, C=C and C=O indicates that the proteins and polysaccharides could be capping and stabilizing agents, whereas the fatty acids could be responsible for stabilizing the AgNPs. GC-MS showed that elaidic acid (18.36%) was the most abundant fatty acid in the sample, followed by monoolein (17.37%) and linoelaidic acid (12.83%), suggesting that these fatty acids could be the major stabilizers of AgNPs. We hypothesized that these fatty acids coated the surface of AgNPs. For instance, in the supernatant and D_Sup_@AgNPs, IRs located at 1790 and 1740 cm^−1^, respectively, corresponding to C=O, matched that existence in the elaidic acid range. Also, IRs located at 1380 cm^−1^ were detected in D_Bio_@AgNPs and D_Sup_@AgNPs, corresponding to the C–H of elaidic acid. The IR peaks at 2855, and 2926 cm^−1^ correspond to the C–H of linoelaidic acid surrounding NPs. These data agree with Mudhaffar et al., who reported the IRs of pure linoelaidic acid at 2927 and 2855 cm^−1^ [41]. Lu et al. coated cobalt NPs with oleic and elaidic acids and found that the IR peaks of the pure acids were located at 1715 cm^−1^, corresponding to C=O, while it was shifted to be 1550 cm^−1^ in NPs after being coated with fatty acids. The authors detected a board peak at 683–611 cm^−1^, referring to the CH=CH of both acids [42]. However, further studies are required to explore the exact mechanism of AgNP synthesis using *D. edaphica* extracts. Mora-Godínez et al. synthesized AgNPs using cell pellets, cell-free medium, and cell pellets with a free medium of *Desmodesmusabundans* at low (LCA) and high (HCA) carbon acclimation [43]. The authors found that the surface of the AgNPs was composed of amides (N–H), polysaccharides (CH_2_ and CH_3_), and fatty acids (C=O and O–H), which were the main capping agents during the synthesis process.

### 2.3. Antiproliferative Activity of D_Bio_@AgNPs and D_Sup_@AgNPs

**Figure 9:**
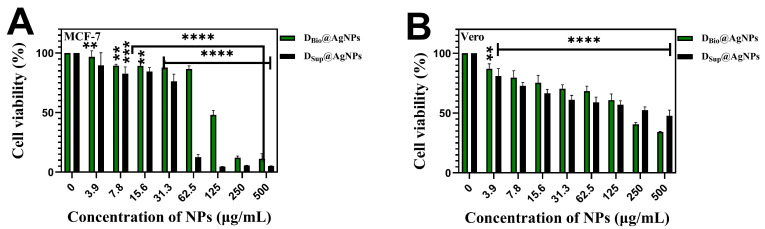
Cell viability of MCF-7 (**A**) and Vero (**B**) cells before and after being treated with D_Bio_@AgNPs and D_Sup_@AgNPs synthesized using *D. edaphica* biomass extract and cell free medium, respectively. **** *p* < 0.0001, *** *p* < 0.0001, and ** *p* < 0.005.

The antiproliferative activities of D_Bio_@AgNPs and D_Sup_@AgNPs against breast cancer cells (MCF-7) and normal kidney cells derived from African green monkey (Vero) were studied using the MTT assay (Figure 9). The results revealed that D_Bio_@AgNPs and D_Sup_@AgNPs significantly reduced the cell viability in a dose-dependent manner. At the highest concentration of D_Bio_@AgNPs and D_Sup_@AgNPs (500 µg/mL), the MCF-7 cells viability was reduced to 11.2% and 5.2%, respectively, whereas 34% and 47.6% of viable Vero cells was detected at 500 µg/mL of D_Bio_@AgNPs and D_Sup_@AgNPs, respectively. These results reveal that D_Sup_@AgNPs are more potent antiproliferative agents than D_Bio_@AgNPs. Among the two NPs, D_Sup_@AgNPs showed more selectivity toward malignant cells than toward normal cells; consequently, they could be more biocompatible agents toward normal cells. This could be attributed to the influence of the AgNP size on the biological activities. Small AgNPs have a large surface area in contact with the plasma membrane, enabling more Ag ions to enter cells and cause cellular disruption [44]. Moreover, the chemical composition and concentration of the functional groups of the algal coronae surrounding the NPs surface can interfere with their cellular internalization mechanism and consequently impact their antiproliferative activity [19]. The colloidal stability of NPs and their tendency to form aggregates in the cellular environment are other factors that influence their toxicity [45]. More studies are required to understand the mechanism of toxicity of D_Bio_@AgNPs and D_Sup_@AgNPs against MCF-7 and Vero cells. Park et al. reported that small AgNPs (20 nm) showed significantly higher metabolic toxicity against both L929 fibroblasts and RAW 264.7 macrophages than larger AgNPs (80 and 113 nm) [46].

### 2.4. Inhibitory Activity of D_Bio_@AgNPs and D_Sup_@AgNPs against Bacterial Cells

**Figure 10:**
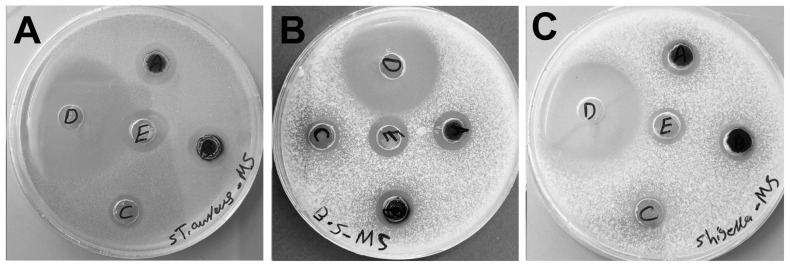
Inhibitory activity of ciprofloxacin (5 μg/mL), D_Bio_@AgNPs, D_Sup_@AgNPs, and Chem@AgNPs (1000 μg/mL) against *Staphylococcus aureus* (**A**), *Bacillus subtilis* (**B**), and *Shigella flexneri* (**C**). Handwritten letters on the petri dishes refer to A: D_Bio_@AgNPs, B: D_Sup_@AgNPs, C: Chem@AgNPs, D: ciprofloxacin, and E: AgNO_3_.

Pathogenic bacteria such as *Staphylococcus aureus* are among the common Gram-positive bacteria that cause widespread skin and respiratory clinical infectious diseases, whereas *Shigella flexneri* is a Gram-negative facultatively intracellular pathogen responsible for bacillary dysentery in humans [47,48]. *Bacillus subtilis* is a well-characterized, non-pathogenic model organism used in microbiological and biochemical studies [49]. Due the tendency of the bacteria to acquire antibiotic resistance, discovering new drugs is a necessity. The biocidal activities of 1 mg/mL of D_Bio_@AgNPs and D_Sup_@AgNPs were examined against *S. aureus*, *B. subtilis*, and *S. flexneri* for 24 h using the agar well diffusion and resazurin-based microdilution methods (Figure 10 and Table 4). D_Bio_@AgNPs and D_Sup_@AgNPs showed excellent biocidal activity against Gram-positive and Gram-negative bacteria; however, their activity was higher against *S. aureus* and *B. subtilis* than against *S. flexneri*. The biocidal activities of ciprofloxacin, D_Bio_@AgNPs, D_Sup_@AgNPs, AgNO_3_, and Chem@AgNPs against the tested bacteria are shown in Table 4. In the order of decreasing biocidal activity, they can be arranged as follows: ciprofloxacin > biogenic AgNPs > AgNO_3_ > Chem@AgNPs. Notably, the inhibitory activity of Chem@AgNPs was weak against *S. aureus* and *B. subtilis* and was zero against *S. flexneri* compared with that of biogenic AgNPs. Silver nitrate showed an inhibitory effect against the tested bacteria; however, it was less than that of biogenic AgNPs against *S. aureus* and *B. subtilis* and similar to that of biogenic AgNPs against *S. flexneri.* The more significant biocidal effect of D_Bio_@AgNPs and D_Sup_@AgNPs against the tested bacteria compared with that of Chem@AgNPs could be attributed to the chemical nature of the biomolecules surrounding the AgNPs, which could influence their biocidal activity by facilitating the cellular uptake of these NPs through their interaction with biomembranes. These data suggest that algal corona-surrounded AgNPs could enhance their biocidal potential. AgNPs were found to be more effective against Gram-negative bacteria than against Gram-positive bacteria. The cell wall of Gram-negative bacteria has a thick lipopolysaccharide (LPS) layer and a thin peptidoglycan (PG) layer, whereas Gram-positive bacteria have thin LPS and thick PG layers, which could affect the cellular uptake of AgNPs and, consequently, their biocidal activity [50,51]. Moreover, the positively charged AgNPs showed greater bacterial activity than the negatively charged AgNPs. This could be attributed to the theory that as positively charged ions, silver is readily attracted to negatively charged molecules such as sulfur, phosphorus, and proteins of cell membranes, which could increase the cellular uptake and biocidal activity [51,52]. Our data revealed a significant difference from prior investigations, indicating that *S. aureus* was the bacteria most susceptible to D_Bio_@AgNPs and D_Sup_@AgNPs, followed by *B. subtilis* and *S. flexneri*. This represents another significant advantage of our biogenic AgNPs in terms of the biocompatibility against mammalian cells, assuming that cationic NPs are more toxic than those with neutral or negative surface charges [53,54]. Additionally, we believe that the high toxicity of D_Bio_@AgNPs and D_Sup_@AgNPs against Gram-positive bacteria could be attributed to the negatively charged functional groups on the AgNP surfaces, which facilitate the entry of these NPs through unknown specific mechanisms. Additionally, this could be related to the nature of the bacterial strain (resistance potential, metabolic activity, and cellular mechanisms), which could interfere with AgNP uptake [55]. Further studies are required to investigate the influence of the algal corona on NP cellular uptake. Kong et al. studied the effect of AgNP size on multiple biological systems [56]. They have shown that smaller AgNPs exert greater toxicity than large ones against bacterial activities such as bioluminescence, enzymes, and enzyme biosynthesis.

## 3. Material and Methods

### 3.1. Algal Isolation, Culture, and Identification

The algae used in this study was isolated from soil samples collected in Riyadh, Saudi Arabia. The samples were transferred to the lab and kept in sterilized Petri dishes containing BG-11 media under illumination (2000 ± 200 lx) in an incubator for 1 week. Later, the samples were purified according to the method described by Bolch et al. [57], as presented in a previous study [58]. The purity of the samples was examined under a light microscope. The purified sample was then cultured in a 1000 mL flask under the same growth conditions but with additional agitation using an air pump. The samples were morphologically identified using a light microscope. To genetically identify the sample, the genomic DNA was extracted using a kit (Attogene, Austin, TX, USA) according to the manufacturer’s instructions. PCR was used to amplify the sample using the forward primer CCTGGTTGATCCTGCCAG and reverse primer TTGATCCTTCTGCAGGTTCA [59]. The amplicons were sent to Macrogen for 18s rRNA sequencing. The phylogenetic tree was constructed using the cluster method and MEGA4 software version 10.2.6. The number at each branch refers to the bootstrap values for the % of 1000 replicate trees calculated by the neighbor-joining statistical method.

### 3.2. Algal Extract Preparation and Phytochemical Content Analysis

On day 15 of the algal culture, the microalgae were centrifuged at 4700 rpm for 10 min to collect the cell-free medium, which was filtered using Whatman filter paper No. 1. The filtrate (solution 1, referred to as D_Sup_) was used to synthesize AgNPs. The biomass was washed at least thrice with distilled H_2_O and freeze-dried using LYOTRAP (LTE Scientific, Greenfield, UK) for 24 h. The dried biomass was mixed with glass beads, vortexed, and crushed to a fine powder. The algal biomass extract was obtained by mixing 200 mg of the algal powder with 100 mL of distilled H_2_O and boiled in a water bath at 60 °C for 30 min. The mixture was cooled to room temperature and centrifuged at 4700 rpm for 10 min. The supernatant was filtered using Whatman filter paper No. 1 to obtain the final filtrate (solution 2: D_Bio_), which was used to synthesize the AgNPs. Notably, the volatile biomolecules in the algal biomass extract were analyzed using Trace GC-TSQ mass spectrometer (Thermo Scientific, Austin, TX, USA) with a direct capillary column TG−5MS (30 m × 0.25 mm × 0.25 μm film thickness) according to the method described in our previous study [19].

### 3.3. Green Synthesis of AgNPs Using Algal Biomass and Cell-Free Medium Using D. edaphica and Physicochemical Characterizations

#### 3.3.1. Optimization of AgNP Synthesis Using Microalgal Biomass and Supernatant

We extracellularly synthesized AgNPs using the algal *D. edaphica* strain CCAP 6006/5 biomass extract (D_Bio_) and cell-free medium (D_Sup_) by controlling the reaction parameters, such as silver nitrate concentration, the algal extract/silver nitrate (V_mL_/V_mL_) ratios, temperature, reaction time, illumination, and pH. To study the effect of various AgNO_3_ concentrations on the synthesis reaction involving D_Bio_@AgNPs and D_Sup_@AgNPs, 1 mL of algal extract was mixed with 2 mL of 1, 2, 4, and 8 mM of AgNO_3_. All the other reaction conditions (25 °C, light exposure, pH initial without adjustment for 24 h) were kept constant. After 24 h of incubation, the wavelengths of the NP suspension aliquots were measured at each concentration using UV-VIS spectroscopy (Model UV-1800, Shimadzu, Kyoto, Japan). The optimum concentrations were determined based on the lowest AgNP wavelength value in the range of 400–460 nm. The effect of the algal extract/silver nitrate (V_mL_/V_mL_) ratio on the AgNP synthesis process under constant reaction conditions (2 mM of AgNO_3_, 25 °C, light exposure, pH initial without adjustment for 24 h) was determined by mixing the algal extracts (D_Bio_ and D_Sup_) and AgNO_3_ at various ratios of 1:1, 1:2A, 1:4A, 1:9A, 1:2B, 1:4B, and 1:9B. The letter A in the algal extract/AgNO_3_ ratio indicates that the volume of AgNO_3_ was changed against that of the of algal extract, which was kept constant, whereas the letter B indicated that the algal extract volume was changed and that of AgNO_3_ was kept constant. Next, the algal extracts were mixed with 2 mM AgNO_3_ at optimum ratios and the mixtures were incubated at 40, 60, 80, and 100 °C in a water bath for 1 h while the other reaction conditions were kept constant. After 1 h, all the mixtures were incubated under illumination for 24 h. The effect of the reaction time was determined by exposing the algal extract/precursor mixture to the optimum temperature for 15, 30, and 60 min under constant reaction conditions. The experiment was performed once in the dark and once in the light under optimal conditions after mixing the algal extracts with AgNO_3_. The effect of the pH was studied by modifying the initial pH of the algal extract/AgNO_3_ mixture using 1 M HCL or NaOH to 6, 7, 8, and 12 under optimum synthesis reaction conditions. Finally, D_Bio_@AgNPs and D_Sup_@AgNPs were synthesized on a larger scale using the following optimum parameters: D_Bio_@AgNPs: mixing 1 mL algal extract with 9 mL of 2 mM AgNO_3_ at 25 °C under illumination and pH 7 for 24 h; D_Sup_@AgNPs: mixing 1 mL algal extract with 2 mL of 2 mM AgNO_3_ at 60 °C for 1 h and under illumination and pH 9.5 for 24 h [19].

To determine the size and shape of the synthesized D_Bio_@AgNPs and D_Sup_@AgNPs, the samples were examined using transmission and scanning electron microscopy. In brief, 1 mL of the AgNP suspension was centrifuged at 13,000× *g* rpm for 15 min and washed at least thrice with distilled H_2_O. The samples were suspended in 1 mL water and sonicated for 15 min in a water bath. Then, AgNP suspension drops (10 µL/drop) were placed on carbon-coated copper grids (300 mesh) and air-died in a laminar flow. The samples were examined using TEM (JEM-1400Flash, Jeol, Akishima, Japan) at 120 kV. Similarly, 10 µL of AgNP suspensions was dropped onto a glass piece embedded in a copper stub and coated with platinum using a coater. The samples were examined using SEM (JEC-3000FC, Jeol, Japan) at 15 kV. To determine the elemental composition and distribution of D_Bio_@AgNPs and D_Sup_@AgNPs, the samples were dried using a lyophilizer, and a small quantity of each AgNP powder was transferred to a carbon strip attached to the SEM stub for examination using an EDx detector conjugated to the SEM. The hydrodynamic diameters and potential charges of the D_Bio_@AgNP and D_Sup_@AgNP aqueous suspensions were determined using a Zetasizer (Malvern, UK). FTIR spectroscopy (Shimadzu, Kyoto, Japan) was performed in the range of 400–4000 cm^−1^ to study the surface chemistry of D_Bio_@AgNPs and D_Sup_@AgNPs (in powder form) and elucidate the interaction between the algal biomolecules and Ag ions. Notably, the functional groups of the biomolecules present in the algal biomass extract and cell-free medium were detected [19].

#### 3.3.2. Anticancer Activity of D_Bio_@AgNPs and D_Sup_@AgNPs

The antiproliferative and biocompatibility effects of 500 µg/mL of D_Bio_@AgNPs and D_Sup_@AgNPs were evaluated against human breast cancer cells (MCF-7) and normal kidney cells of African green monkey (Vero) that were grown on complete DMEM medium (10% fetal bovine serum and 50 U/mL of penicillin and streptomycin) in an incubator at 37 °C and 5% CO_2_. After 75% confluency was reached, the cells were passaged using trypsin–EDTA, counted, seeded (5 × 10^3^ cells/well) into 96-well plates, and incubated for 24 h under the same conditions. For the D_Bio_@AgNPs and D_Sup_@AgNPs suspension preparation: 1 mg of D_Bio_@AgNPs and D_Sup_@AgNPs were mixed with 1 mL of DMEM and sonicated for 15 min until well dispersed in the medium. The samples were filtrated using 0.45 µm sterilized syringe filter. Then, 2-fold serial dilution samples (500, 250, 125, 62.5, 31.25, 15.6, 7.8, and 3.9 µg/mL) were prepared and applied to the cells, followed by incubation for 24 h. Then, the old media were replaced with fresh media, and 10 µL of MTT solution (5 mg/mL) was added to the cells, which were kept for 4 h in an incubator. Then, the media were discarded and 100 µL/well of DMSO was added. The plates were kept for 15 min. The optical absorbance of the sample was measured at 570 nm, and the cell viability % was estimated using the following equation [19,60]:

(Abs(treated)/(Abs(control)) × 100

#### 3.3.3. Antibacterial Activity of D_Bio_@AgNPs and D_Sup_@AgNPs

The inhibitory effect of 1000 µg/mL of D_Bio_@AgNPs and D_Sup_@AgNPs was detected against *Staphylococcus aureus* ATCC 29213, *Bacillus subtilis* ATCC6633, and *Shigella flexneri* (clinical isolate) using the agar well diffusion method. The bacterial strains were grown in nutrient broth for up to 18 h at 37 °C and maintained by continuous subculturing in broth and agar media. Nutrient agar plates of bacterial suspension (2.5–3.6 × 10^6^ CFU/mL) were prepared in 8 mm diameter wells. The bacteria were treated with 100 μL of 1000 µg/mL of D_Bio_@AgNPs and D_Sup_@AgNPs suspensions (sonicated for 15 min before treatment) and kept in the incubator at 37 °C for 24 h. Notably, ciprofloxacin (5 µg/mL) was used as a positive control. The diameters of the inhibition zones (in mm) were measured using a transparent ruler.

The minimum inhibitory concentrations (MIC) of 500 µg/mL D_Bio_@AgNPs and D_Sup_@AgNPs were determined using the resazurin dye method described by Elshikh et al. [61]. Serial dilution concentrations of D_Bio_@AgNPs and D_Sup_@AgNPs (500, 250, 125, 62.5, 31.25, 15.62, 7.8, 3.9, 1.95, and 0.98 µg/mL) were prepared and 100 µL of NP suspension was mixed with 100 µL of bacterial suspension (2.5–3.6 × 10^6^ CFU/mL) in 96-well plates. Two control groups were established: a positive control group containing an untreated bacterial suspension and a negative control group consisting of medium only to assess sterility. The plates were left to culture overnight at a temperature of 37 °C. Following incubation, the bacteria were incubated with 30 µL of a 0.015% resazurin dye solution at a temperature of 37 °C for 4 h. The absorbance of the samples was quantified at 570 nm using a plate reader (Hercules, CA, USA). After 4 h, the column was considered above the MIC value if there was no color change (the color of the resazurin indicator, blue, remained unchanged).

### 3.4. Statistical Analysis

GraphPad Prism v.9.3.1 (GraphPad Software Inc., San Diego, CA, USA), Origin 8 (OriginLab Corporation, Northampton, MA, USA), and ImageJ 1.52a (National Institutes of Health, Bethesda, MD, USA) software were used to analyze the data in the current study. The data were presented as the mean ± standard error of the mean (SEM). Significance was detected using a one-way analysis of variance (ANOVA). The data were considered statistically significant at a *p* value < 0.05.

## 4. Conclusions

To the best of our knowledge, the current study is the first to represent the potential of the novel *D. edaphica* strain CCAP 6006/5 to biosynthesize AgNPs using its biomass extract and cell-free medium and to report the phytochemical compositions of these microalgae using GC-MS. Moreover, this study focused on determining the optimum reaction parameters to synthesize small, stable, and well-dispersed AgNPs by controlling parameters such as the AgNO_3_ concentration, algal/silver nitrate ratio, temperature, reaction time, illumination, and pH. The results showed that *D. edaphica* biomass extract and cell-free medium have great bioreduction potential to form small-sized D_Bio_@AgNPs and D_Sup_@AgNPs. Several physicochemical parameters, such as the AgNO_3_ concentration, algal/silver nitrate ratio, temperature, reaction time, illumination, and pH, influence the size and stability of these NPs. This indicates that during the biological synthesis of NPs using microalgae, the reaction must be optimized to regulate their physicochemical characteristics. D_Bio_@AgNPs and D_Sup_@AgNPs present quasi-spherical shapes, with small sizes of 15.0 ± 1.0 nm and 12.0 ± 0.8 nm, respectively. The HDs of D_Bio_@AgNPs and D_Sup_@AgNPs were 77.9 and 62.7 nm and the charges were −24.4 and −25.8 mV, respectively. This negativity on the NP surface could be attributed to the algal corona surrounding the AgNPs. The surface of these biogenic AgNPs are coated with various functional groups, including O–H, C–H, C=C/N–H, C–O, C=O, and C–Br, suggesting that algal polysaccharides and proteins operate as reductants, whereas polysaccharides and fatty acids act as stabilizers during NP formation. The GC-MS analysis revealed that elaidic acid (18.36%) and monoolein (17.37%) were the most abundant biomolecules in the algal extracts, indicating that these fatty acids may affect AgNP stabilization. D_Sup_@AgNPs showed more potent anticancer activity against MCF-7 cells and better biocompatibility with Vero cells than D_Bio_@AgNPs. In contrast, D_Bio_@AgNPs and D_Sup_@AgNPs showed greater biocidal activity against Gram-negative and Gram-positive bacteria than Chem@AgNPs and AgNO_3_. These results indicate that *D. edaphica* strain CCAP 6006/5 is a facile and eco-friendly biomachine for the synthesis of AgNPs and may be used as a powerful therapeutic agent against cancer and infectious diseases.

## Figures and Tables

**Table 1:** Phytochemicals detected in the *Desmochloris edaphica* strain CCAP 6006/5 biomass extract using gas chromatography–mass spectrometry (GC-MS).

No.	Compound	Retention Time	Area%	Matched Factor	Molecular Formula	Molecular Weight
1	Methyl jasmonate	19.32	0.76	901	C_13_H_22_O_3_	226
2	Acetic acid, [1-(4-isopropylphenyl)-2-methyl]propyl ester	19.86, 19.98	5.60, 1.01	898, 886	C_15_H_22_O_2_	234
3	3-methyl-4-(2,6,6-trimethyl-2-cyclohexen-1-yl)-3-buten-2-one	20.51	1.29	835	C_14_H_22_O	206
4	Versalide	23.65	3.50	917	C_18_H_26_O	258
5	Methyl linolenate	25.54	3.69	919	C_19_H_32_O_2_	292
6	Palmitic acid	26.21	5.95	925	C_16_H_32_O_2_	256
7	9,11-octadecadienoic acid, methyl ester, (9Z,11E)-	28.53	1.27	919	C_19_H_34_O_2_	294
8	Methyl vaccenate	28.71	1.83	905	C_19_H_36_O_2_	296
9	Linoelaidic acid	29.25	12.83	936	C_18_H_32_O_2_	280
10	Elaidic acid	29.44	18.36	939	C_18_H_34_O_2_	282
11	Stearic acid	29.89	2.50	910	C_18_H_36_O_2_	284
12	Glycidyl palmitate	31.91	0.89	862	C_19_H_36_O_3_	312
13	Glyceryl linolenate	34.56	3.45	823	C_21_H_36_O_4_	352
14	Glycidyl oleate	34.70	4.50	906	C_21_H_38_O_3_	338
15	2-palmitoylglycerol	35.08	1.59	892	C_19_H_38_O_4_	330
16	1,2-dipalmitin	35.19	1.05	796	C_35_H_68_O_5_	568
17	Diisooctyl phthalate	35.68	0.79	932	C_24_H_38_O_4_	390
18	2-monolinolein	37.61	9.24	893	C_21_H_38_O_4_	354
19	Monoolein	37.73	17.37	880	C_21_H_40_O_4_	356
20	2-monostearin	38.17	1.60	825	C_21_H_42_O_4_	358
21	Arabinitol, pentaacetate	45.01	0.92	728	C_15_H_22_O_10_	362

**Table 2:** Elemental composition of D_Bio_@AgNPs and D_Sup_@AgNPs synthesized using *D. edaphica* biomass extract and cell-free medium, respectively.

D_Bio_@AgNPs				D_Sup_@AgNPs			
Element	Line	Mass%	Atom%	Element	Line	Mass%	Atom%
C	K	6.72 ± 1.41	34.72 ± 0.28	O	K	1.54 ± 0.02	7.64 ± 0.10
O	K	1.46 ± 0.94	5.67 ± 0.14	Al	K	0.27 ± 0.01	0.79 ± 0.03
Al	K	0.21 ± 0.22	0.48 ± 0.02	Si	K	0.60 ± 0.01	1.69 ± 0.03
Cl	K	4.30 ± 0.41	7.53 ± 0.03	Cl	K	11.82 ± 0.03	26.45 ± 0.06
Cu	K	1.99 ± 0.66	1.95 ± 0.03	Cu	K	0.63 ± 0.04	0.78 ± 0.05
Zn	K	1.51 ± 0.72	1.43 ± 0.03	Ag	L	85.15 ± 0.11	62.66 ± 0.08
Ag	L	83.81 ± 2.29	48.22 ± 0.05	Total		100.00	100.00
Total		100.00	100.00				

**Table 3:** Functional groups in the biomass extract and cell-free medium and surrounding the D_Bio_@AgNPs and D_Sup_@AgNPs synthesized using *D. edaphica* biomass extract and cell-free medium, respectively.

Biomass Extract		D_Bio_@AgNPs		Cell Free Medium Extract		D_Sup_@AgNPs	
FTIR (cm^−1^)	Functional Group	FTIR (cm^−1^)	Functional Group	FTIR (cm^−1^)	Functional Group	FTIR (cm^−1^)	Functional Group
3346	O–H	3437	O–H	3366	O–H	3437	O–H
2937	C–H	2926	C–H	2937	C–H	2926	C–H
2849	C–H	2855	C–H	2849	C–H	2855	C–H
1561	N–H/C=C	1750	C=O	2435	N–H	1740	C=O
1400	C–H	1632	C=C/N–H	1790	C=O	1622	N–H/C=C
1116	C–O	1461	C–H	1632	C=C/N–H	1461	C–H
1030	C–O	1380	C–H	1349	C–H	1380	C–H
928	C–H	1060	C–O	1146	C–O	1050	C–O
867	C–H	883	C–H	1045	C–O	766	C–H
782	C–H	528	C–Br	878	C–H	573	C–Br
659	C–Br			837	C–H		
622	C–Br			782	C–H		
544	C–Br			622	C–Br		

**Table 4:** MIC (µg/mL) of D_Bio_@AgNPs and D_Sup_@AgNPs and inhibition zone (mm) of ciprofloxacin, D_Bio_@AgNPs, D_Sup_@AgNPs, Chem@AgNPs, and AgNO_3_ against *Staphylococcus aureus*, *Bacillus subtilis*, and *Shigella flexneri*.

Drugs	* S. aureus *	* B. subtilis *	*S. flexneri*
** MIC (µg/mL) **			
D_Bio_@AgNPs	62.5	62.5	62.5
D_Sup_@AgNPs	3.9	31.3	3.9
** IZ (mm) **			
D_Bio_@AgNPs	17.7 ± 0.1	17.1 ± 0.03	14.6 ± 0.1
D_Sup_@AgNPs	16.8 ± 0.1	16.02 ± 0.06	14.9 ± 0.05
AgNO_3_	15.7 ± 0.03	15.9 ± 0.05	14.6 ± 0.1
Chem@AgNPs	12.1 ± 0.1	10.1 ± 0.2	0.0 ± 0.0
Ciprofloxacin	32.3 ± 0.4	33.1 ± 0.1	30.5 ± 0.1

## Data Availability

Additional data to those presented here are available from the corresponding author upon reasonable request.
